# Donor-host mitochondrial compatibility improves efficiency of bovine somatic cell nuclear transfer

**DOI:** 10.1186/1471-213X-10-31

**Published:** 2010-03-19

**Authors:** Zhong-hai Yan, Yi-ye Zhou, Jing Fu, Fei Jiao, Lei-wen Zhao, Peng-fei Guan, Shu-zhen Huang, Yi-tao Zeng, Fanyi Zeng

**Affiliations:** 1 Shanghai Institute of Medical Genetics, Shanghai Children's Hospital, Shanghai Jiao Tong University School of Medicine, Shanghai, PR China; 2 Institute of Medical Science, Shanghai Jiao Tong University School of Medicine, Shanghai, PR China

## Abstract

**Background:**

The interaction between the karyoplast and cytoplast plays an important role in the efficiency of somatic cell nuclear transfer (SCNT), but the underlying mechanism remains unclear. It is generally accepted that in nuclear transfer embryos, the reprogramming of gene expression is induced by epigenetic mechanisms and does not involve modifications of DNA sequences. In cattle, oocytes with various mitochondrial DNA (mtDNA) haplotypes usually have different ATP content and can further affect the efficiency of *in vitro *production of embryos. As mtDNA comes from the recipient oocyte during SCNT and is regulated by genes in the donor nucleus, it is a perfect model to investigate the interaction between donor nuclei and host oocytes in SCNT.

**Results:**

We investigated whether the *in vitro *development of reconstructed bovine embryos produced by SCNT would be influenced by mtDNA haplotype compatibility between the oocytes and donor cells. Embryos from homotype A-A or B-B showed significantly higher developmental ability at blastocyst stages than the heterotype A-B or B-A combinations. Post-implantation development ability, pregnancy rate up to day 90 of gestation, as well as percent of term births were higher in the homotype SCNT groups than in the heterotype groups. In addition, homotype and heterotype SCNT embryos showed different methylation patterns of histone 3-lysine 9 (H3K9) genome-wide and at pluripotency-related genes (*Oct-4, Sox-2, Nanog*).

**Conclusion:**

Both histone and DNA methylation show that homotype SCNT blastocysts have a more successful epigenetic asymmetry pattern than heterotype SCNT blastocysts, which indicates more complete nuclear reprogramming. This may result from variability in their epigenetic patterns and responses to nuclear reprogramming. This suggests that the compatibility of mtDNA haplotypes between donor cells and host oocytes can significantly affect the developmental competence of reconstructed embryos in SCNT, and may include an epigenetic mechanism.

## Background

Although nuclear transfer has been applied to a range of species [1], embryos generated via somatic cell nuclear transfer (SCNT) generally have low developmental competency and many abnormalities occur in the course of development. The underlying mechanisms for these limitations are unclear. Many recent studies on nuclear transfer embryos revealed that gene expression patterns in the cloned embryo, fetus and placenta were abnormal [2] and suggested that these commonly observed abnormalities were attributed to inefficient or incomplete "nuclear reprogramming". It is generally accepted that in nuclear transfer embryos, the reprogramming of gene expression is linked to epigenetic mechanisms and does not involve modification of DNA sequences. Epigenetic modifications such as DNA methylation of CpG islands, core histone acetylation, phosphorylation, ubiquitination and ribosylation can eventually alter the transcriptional status of individual genes or larger genomic regions. Particularly, for the normal development of embryos, proper modifications of both DNA (by methylation) and histones are necessary. On one hand, DNA methylation can lead to gene silencing and heterochromatin formation [3,4] such that proper DNA methylation patterns are crucial in the early development of postimplantation embryos, especially for some key reprogramming factors such as *Oct3/4, Sox2 *and *Nanog *normally activated in the blastocyst stage [3]. On the other hand, histone modifications, which often are directly associated with DNA methylation patterns, also play an important role in heterochromatin formation, mitosis, and regulation of genes and regional chromosome structure. Methylation of histone 3 lysine 9 (H3K9) and histone 3 lysine 27 (H3K27) residues correlate with gene silencing by chromatin condensation [4,5]. In nuclear transfer embryos, abnormal patterns of methylation for DNA and H3K9 have been reported [6].

On a more cellular level, the interaction between the donor nucleus and recipient cytoplast may influence a number of important biological functions in SCNT during nuclear reprogramming. When considering the karyoplast-cytoplast interaction, mitochondria are the most abundant organelle in cytoplasm and play an important role in development by supplying energy for normal cellular functions. Mitochondrial DNA (mtDNA) is supplied mainly by the recipient oocyte during SCNT, but is regulated by genes in the donor nucleus. Under the high oxygen environment, and with limited DNA repair ability, mtDNA has high rates of heritable polymorphism and *de novo *mutation which can result in many haplotypes. In cattle, oocytes with various mtDNA haplotypes usually have different ATP content and this may affect the efficiency of *in vitro *production of embryos [7-9]. However, the relationship and the underlying mechanisms between mtDNA haplotypes and SCNT efficiency have not been fully investigated.

In this study, we divided bovine mtDNA into four haplotypes by PCR-RFLP (Restriction Fragment Length Polymorphism) with six restriction sites. Subsequently, SCNT was performed using donor nuclei and recipient oocytes with particular mtDNA haplotypes to compare developmental competency for different haplotype combinations. When the donor cells and oocytes are of the same mtDNA haplotype (termed homotype SCNT), higher SCNT embryo survival rates were observed. To further explore the underlying mechanisms, the status of homotype and heterotype SCNT blastocysts, *in vitro *fertilization (IVF) blastocysts and *in vivo *cultured (IVC) blastocysts were compared using their histone and DNA methylation patterns. Genome-wide H3K9 dimethylation profiles were detected by immunohistochemistry, and DNA methylation status at the promoters of pluripotency genes *Oct-4, Nanog *and *Sox2 *was also analyzed. Low methylation levels in the promoter regions of these genes is thought to correlate with their high expression levels at the blastocyst stage. Both histone and DNA methylation show that homotype SCNT blastocysts have an epigenetic status closer to the control IVF embryos than heterotype SCNT blastocysts. This study indicates that homotype SCNT has a more complete nuclear reprogramming than heterotype SCNT and, therefore, is more competent for further embryonic development.

## Results

### Ovum Pick-Up (OPU)

**Figure 1:**
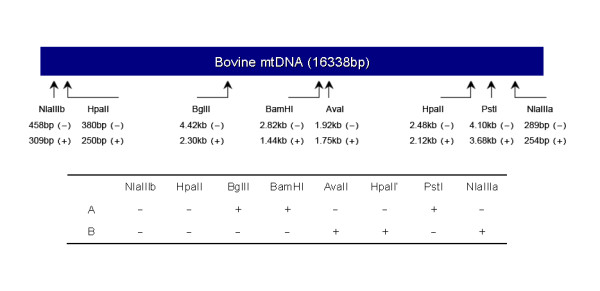
**mtDNA haplotypes determined by different restriction sites and frequencies of A and B types are noted (n > 500)**.

Four mtDNA haplotypes were identified in research herds of Holstein and Chinese Yellow cattle (Fig 1). Two haplotypes, A and B, were chosen for preparation of donor nuclei and recipient oocytes as they represented 25% and 53%, respectively of several hundred cattle tested previously. The average number of oocytes recovered per heifer was 9.3 cumulus-oocyte complexes (COCs). A total of 2625 COCs were obtained from 282 OPU procedures. 2103 high-quality MII oocytes were used for SCNT and 522 for IVF.

### Pre- and Post-implantation Development of Embryos from Homotype and Heterotype SCNT

As summarized in Table 1, after the 8-cell stage, homotype SCNT embryos constructed from enucleated oocytes and donor cells with the same haplotype (A-A or B-B) had significantly higher developmental potential than heterotype SCNT embryos (A-B or B-A), as well as a higher blastocyst rate (42.5% vs. 28.8%) which is similar to rates from IVF embryos (40% based on cleavage number) [10]. Note that the homotype SCNT, heterotype SCNT and IVF embryos were produced from the same OPU session, and the procedures and assays for the three groups were kept as similar as possible. Differences in pregnancy rate at 20, 60 and 90 days of gestation were also observed between homotype and heterotype groups. One hundred and three homotype SCNT blastocysts and 70 heterotype SCNT were transferred into 64 and 43 synchronous recipients, and resulted in 42.2% and 23.3% 90-day pregnancy rates for homotype and heterotype, respectively. The percentage of live term birth for homotype and heterotype groups were 17.2% and 9.3%, respectively. Overall, the differences in postimplantation development were statistically significant starting at 60 days after implantation (Table 2).

**Table 1:** *In vitro *preimplantation development of bovine embryos reconstructed with adult fibroblast cells.

SCNT type	Homotype (Oocytes-Donor)		Heterotype (Oocytes-Donor)		IVF
	
	A-A	B-B	A-B	B-A	/
Oocytes	497	1608	672	631	229
No. of replicates	14	40	16	15	7
Oocytes maturation (%)	392 (78.9%)^a^	1221 (75.9%)^a^	518 (77.1%)^a^	478 (75.8%)^a^	177 (77.3%)^a^
*KCC	335	963	426	402	/
Fused and cultured	187	626	258	267	/
Cleaved (%)^f^	135 (72.2)^a^	455 (72.7%)^a^	188 (72.9)^a^	184 (68.9)^a^	111
8-cell (%)^g^	100 (74.0)^a^	295 (64.8)^b^	118 (62.8)^b^	103 (56.0)^c^	NA
Blastocyst (%)^g^	63.46.7)^a^	188.41.3)^b^	61.32.4)^c^	46.25.0)^d^	45 (40.5)^b^
		
	251/590 (42.5%)^a^		107/372 (28.8%)^b^		/

*KCC, karyoplast--cytoplast complexes. Values with different superscript letters a, b, c, d differ with statistical significance (P < 0.05 by *t *test). f: Percentage based on number of surviving and fused reconstructed embryos, which were activated and cultured following KCC fusion. g: Percentage based on number of cleaved reconstructed embryos.

**Table 2:** Postimplantation development of homotype and heterotype somatic nuclear transfer bovine embryos.

SCNT type	Homotype (Oocytes-Donor)		Heterotype (Oocytes-Donor)	
	
	A-A	B-B	A-B	B-A
No. of transferred blastocysts	35	68	31	39
No. of recipients	21	43	17	26
Pregnancy at day 20 (%)	14/21 (66.7)^a^	26/43 (60.5)^b^	10/17 (58.8)^c^	14/26 (53.8)^d^
Pregnancy at day 60 (%)	10/21 (47.6)^a^	21/43 (48.8)^a^	7/17 (41.2)^b^	9/26 (34.6)^c^
Pregnancy at day 90 (%)	9/21 (42.9)^a^	18/43 (41.9)^a^	4/17 (23.5)^b^	6/26 (23.1)^b^
Term births (dead)	3/21	5/43	0/17	2/26
Term births (live)	4/21 (19.0)^a^	7/43 (16.3)^b^	2/17 (11.8)^c^	2/26 (7.7)^d^
	
	11/64 (17.2)^a^		4/43 (9.3)^b^	

a, b, c, d values within same horizontal columns with different superscripts differ significantly (P < .05 by *t *test).

### Histone H3K9 Dimethylation

**Figure 2:**
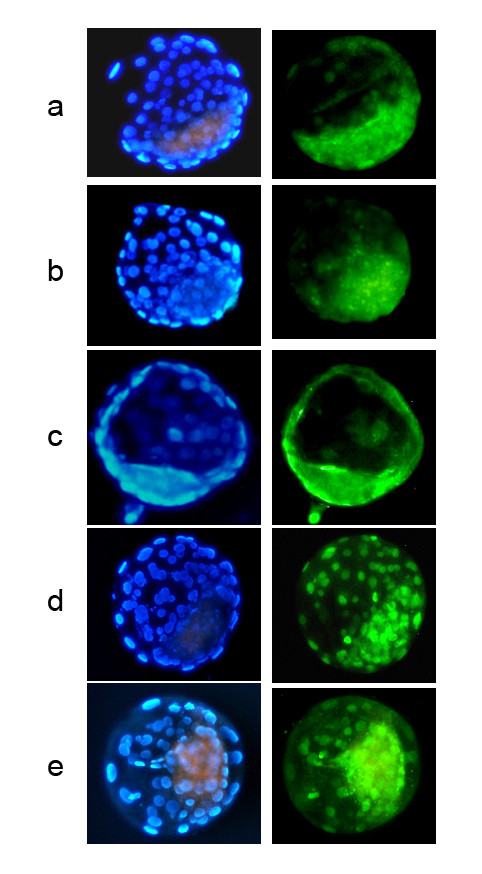
**Epigenetic profiles of homotype SCNT (A-A and B-B), heterotype SCNT (A-B and B-A), and IVF embryos**. The bovine embryos were double stained for histone H3K9 methylation (green) and DNA (blue, stained with DAPI to identify the nuclear compartment). The right column shows representative images of the anti-histone H3-dimethylK9 (H3K9) immunofluorescence from IVF embryos (a, n = 35), A-A SCNT embryos (b, n = 11), B-B SCNT embryos (c, n = 17), A-B SCNT embryos (d, n = 12), and B-A SCNT embryos (e, n = 13). Blastocysts from IVF (a) and homotype SCNT (A-A and B-B) treatments (b, c) showed hypomethylated trophectoderm and hypermethylated ICM, whereas heterotype SCNT (A-B and B-A) blastocysts (d, e) had a more homogeneous pattern between trophectoderm and ICM.

The patterns of distribution and intensity of dimethylated H3K9 were previously shown to closely parallel overall DNA methylation [6]. In our study, H3K9 methylation patterns of homotype SCNT embryos more closely resembled the control IVF embryos than did heterotype SCNT embryos (Fig. 2). In the IVF control, homotype A-A and B-B SCNT groups, 74.3% (26/35), 63.6% (7/11) and 35.3(6/17) of blastocysts respectively established epigenetic asymmetry successfully, with a hypomethylated trophectoderm and a hypermethylated ICM; whereas only 8.3% (1/12) and 7.7%(1/13) heterotype A-B and B-A SCNT groups achieved asymmetry (Table 3).

**Table 3:** Comparison of H3K9 dimethylation patterns *in vitro *fertilization (IVF), homotype SCNT, and heterotype SCNT bovine blastocysts.

Embryo types (Oocytes-Donor)	No.	H3K9 methylation status	
		
		Normal H3K9 methylation patterns: hypomethylated trophectoderm and hypermethylated ICM (%)	Aberrant H3K9 methylation patterns: little difference between ICM and trophectoderm (%)
IVF blastocyst	35	26 (74.3)	9 (25.7)^a^
A-A	11	7 (63.6%)	4 (36.4%)^b^
B-B	17	6 (35.3%)	11 (64.7%)^c^
A-B	12	1 (8.3%)	11 (91.7%)^d^
B-A	13	1 (7.7%)	12 (92.3%)^d^
**Homotype SCNT**	**28**	**13 (46.4)**	**15 (53.6)**^e^
**Heterotype SCNT**	**25**	**2 (8.0)**	**23 (92.0)**^d^

a, b, c, d, e values in same columns with different superscripts differ significantly (P < 0.05) based on Fisher's exact test.

### Methylation Status of Pluripotency Regulators

**Figure 3:**
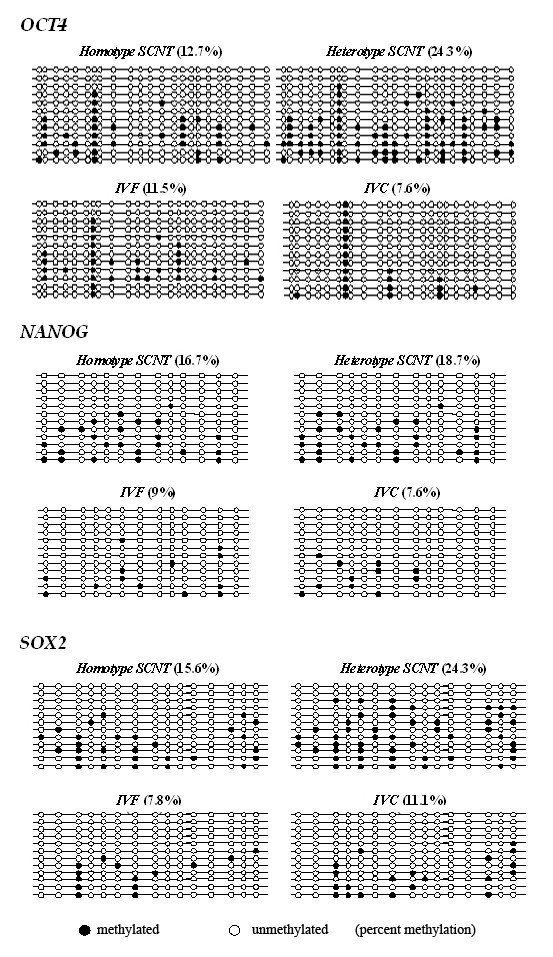
**DNA methylation patterns of pluripotency gene promoters in various embryos**. The results were obtained from three independent DNA samples. Each horizontal row of circles represents an individual sequencing result from one amplicon. Open and filled circles indicate unmethylated and methylated CpG dinucleotides, respectively.

*Oct4, Nanog *and *Sox2 *are known to be critical for the maintenance of pluripotency, and the bisulfite conversion sequencing method was used to examine the methylation patterns of promoter regions of these three genes in normal *in vivo *developed embryos, as well as IVF, homotype SCNT and heterotype SCNT embryos. About 7.6-24.3% of the total CpG sites examined were found to be methylated, and the overall methylation frequencies were different for embryos from the IVF, IVC and NT groups (Table 4). The relative methylation levels at various CpGs for a particular gene seem to be relatively stable within each sample (Fig. 3), implying that the methylation of these promoters in embryos is biased for specific CpG positions. The two SCNT embryos showed various degrees of increased promoter methylation compared to the IVF and IVC groups; however, statistical testing confirmed more significantly increased promoter methylation levels in heterotype SCNT compared to homotype SCNT for *Oct4 *and *Sox2*, but not *Nanog *(Table 4), relative to the control groups.

**Table 4:** Statistical difference tests for promoter DNA methylation.

Embryos	*Oct4 *(%)	*Nanog* (%)	*Sox2* (%)
Homotype	12.7✶	16.7	15.6✶
Heterotype	24.3*	18.7	24.3*
IVF	11.5✶	9*✶	7.8*✶
IVC	7.6*✶	7.6*✶	11.1✶

*: Statistically significant difference compared to the homotype group (p < 0.05) analysed with Chi-square test. ✶ : Statistically significant difference compared to the heterotype group (p < 0.05) analysed with Chi-square test.

## Discussion

Early mammalian embryonic development, spanning fertilization to blastocyst, is a very dynamic process, especially in large animals such as cattle and goats. Numerous studies have reported the development capability of *in vitro *produced (IVP) embryos could be influenced by aberrant gene expression levels and epigenetic reprogramming [10-12]. Six to eight days post fertilization or after reconstruction for SCNT embryos is a critical period when various developmentally important events occur, including the first cleavage division which is critical in determining the quality of subsequent embryo development.

In this study, we investigated whether the *in vitro *development of reconstructed bovine embryos produced by OPU-SCNT technique could be influenced by mtDNA haplotype compatibility between the oocytes and donor cells, and we found that the developmental competency of homotype (A-A or B-B) SCNT appeared to be better than that of heterotype (A-B or B-A) SCNT, especially after the eight-cell stage. This result is in agreement with our previous finding that autologous SCNT embryos have a much improved developmental capacity compared to embryos derived with allogeneic SCNT [13]. With the autologous SCNT, the donor cells and the recipient oocytes were derived from the same cattle and thus had the same mitochondrial genetic background whereas in the allogeneic group, the mitochondria haplotypes were not matched. The percentage of pregnant animals at 90 days of gestation was also higher in the homologous haplotype SCNT embryos compared to the heterogeneous group, indicating a more appropriate microenvironment for further embryonic development, as the nuclear-cytoplasmic compatibility in cloned embryos may prevent abnormality during gestation as previously reported [14,15]. The birth rates of live offspring for A-A, B-B, A-B, and B-A haplotypes were 19.0%, 16.3%, 11.8% and 7.7%, respectively. Thus, from the reconstructed embryos to live calves, the survival rate for the homotype SCNT group was 5.3% of reconstructed embryos, and is a little higher than reported in a previous study (0-4%) [15].

During the development of the reconstructed embryos, nuclear-cytoplasm interactions are necessary for successful reprogramming to ensure the proper activation of pluripotency genes. After the donor nuclei enter the oocytes, the differentiated somatic cell nuclei will be epigenetically rebuilt with an "erase-and-rebuild" strategy to set a new life cycle, and then the genes in dedifferentiated nuclei will be expressed to control embryo development, instead of maternal RNAs and proteins after the EGA (activation of the embryonic genome) period [16-18]. In the present study, we found that the blastocyst formation rates as well as postimplantation development capability for the haplotype of A-A SCNT reconstructed embryos were higher than the other types (P < 0.05) (Table 1). This suggests that the mtDNA haplotype of oocytes, in addition to the nuclear-cytoplasmic compatibility of mitochondria, could be an important factor for the development of cloned embryos, possibly related to mtDNA copy numbers and ATP content in the haplotype A oocyte as previous studies indicated [19-21].

During development and differentiation, somatic nuclei acquire highly specialized DNA and chromatin modifications, i.e. the epigenetic marks, which are thought to result in cellular memory of the differentiated states [22]. This may involve epigenetic changes similar to those that occur in normal embryos during early development [3,23,24]. However, there is accumulating evidence that epigenetic reprogramming is severely deficient in cloned embryos [2,25-27]. Several reports reveal inefficient demethylation and inappropriate reestablishment of DNA *de novo *methylation in quantitative and qualitative patterns during SCNT [2,25,27]. Given that there are potential variations associated with development for the two types of SCNT embryos (Tables 1 and 2), we further analyzed epigenetic modifications for DNA and histones in these embryos. Using Hoechst 33342 and an antibody that identifies H3K9 methylation specifically in the context of constitutive heterochromatin, we double labelled embryonic cells to establish the distribution of H3K9 methylation in IVF control embryos and embryos produced by homotype and heterotype SCNT. Interestingly the H3K9 profile of embryos from homotype SCNT resembles more closely that from the control (IVF) embryos compared to the heterotype SCNT embryos, and only 2 out of 25 heterotype SCNT embryos from B-A and A-B groups showed the correct epigenetic pattern. This suggests that homotype SCNT embryos have fewer H3K9 epigenetic defects than the heterotype SCNT embryos (Table 3, Fig. 2).

DNA methylation/demethylation processes occur during pre-implantation development. Genes such as *Oct4, Klf4, Foxd3 *and *Nanog*, which are required for the maintenance of pluripotency, are inactivated in donor fibroblast cells because of the hypermethylation of their promoters until cloned embryos begin demethylation and development. In the case of *Oct4*, the down-regulation is triggered by trans-acting repressors and the methyl-CpG-binding proteins MBD2 and MBD3 [28]. The locus then gradually becomes "heterochromatinised" followed by the methylation of H3K9, the binding of heterochromatin protein 1 (HP1) beta and DNA methylation [29]. Hypomethylation of regulatory regions of these genes would generally be expected to result in up-regulated expression. We analyzed the methylation profiles for three pluripotency-related genes, *Oct4, Sox2 *and *Nanog *in the homotype and heterotype SCNT (as represented by the A-A and the B-A groups respectively) using *in vitro *and *in vivo *produced blastocysts. Although there are various degrees of increased methylation in the SCNT embryos compared to those from IVF or *in vivo *produced embryos, our data indicated a much more significant change in DNA methylation for *Oct4 *and *Sox2 *for the heterotype compared to homotype SCNT embryos. On the other hand, methylation for *Nanog *was increased for both types of SCNT embryos but with no significant differences between the two groups. Previous studies showed that the initiation of transcriptional activity of the embryonic genome occurs around the transition period from 8-cell to 16-cell for NT embryos [13,30], and the changes in epigenetic modifications occurring during this period (reflected by either global or specific genes' methylation patterns) are critical for the appropriate reprogramming to be initiated. The differences seen in epigenetic status of the SCNT and the IVF embryos, as well as between the homotype and the heterotype embryos, reflects the underlying developmental competence of these embryos; future analysis of factors involved in critical functions in preimplantation development, such as the somatic-type DNA methyltransferase and the dynamic patterns of methylation in H3K9 and pluripotency-related genes, may elucidate the underlying mechanisms governing reprogramming in NT and embryos.

## Conclusion

We report that the compatibility of donor nuclei and recipient oocytes in SCNT is associated with epigenetic modifications which result in different responses to reprogramming. These results may explain why certain mitochondrial DNA haplotypes are inherently more efficient for cloning. Therefore, an approach may be available to improve cloning efficiency by using proper donor-recipient populations to control mitochondrial DNA haplotype combinations.

## Methods

All chemicals and media were purchased from Sigma Chemical Co. (St. Louis, MO) unless otherwise indicated.

### Animals

All Holstein and Chinese Yellow cattle used in this study were raised in Songjiang Experimental Animal Facility, affiliated with the Shanghai Institute of Medical Genetics, China. They were fed with a mixed ration consisting of hay and commercial concentrate pellets. Only those between 12 and 15 months of age (at the beginning of the study) with similar physical conditions were used for our study.

### mtDNA Haplotypes

Template mtDNA was extracted from white blood cells of individual cattle as described by Brown et al. [31]. Six pairs of H/L primers were designed to amplify the entire mtDNA [19]. The resulting six PCR fragments named H1-H6 were subjected to RFLP analysis. PCR was performed as follows: one single denaturation step of 94°C for 5 min followed by 40 cycles at 94°C (45 or 60 sec), 56°C (45 or 60 sec), and 72°C (90 sec or 3 min), and a final extension step of 72°C for 10 min. For RFLP, initially, 16 restriction digestion enzymes (*Nla*III, *Hpa*II, *Bgl*II, *Ava*II, *Bam*HI, *Pst*I, *Eco*RI, *Hpa*I, *Xho*I, *Hph*I, *Apa *I, *Hin*d III, *Sal*I, *Afl *III, *Xba*I, and *Kpa*I) were used to digest the H1-H6 fragments in our prescreening test [19]. Only the first six restriction enzymes generated RFLP patterns representing common polymorphisms (located within H1-H4 fragments) among our cattle (data not shown). Thus, these six enzymes (*Nla*III for H1, *Hpa*II for H2, *Bgl*II for H4, *Ava*II for H3, *Bam*HI for H3, and *Pst*I for H2) were selected for further screening (Fig 1). Restriction digestions were carried out according to manufacturer's recommendations.

### Ovum Pick-Up (OPU)

OPU was carried out twice a week from about 40 selected dairy heifer (Holstein) donors between 12 and 13 months of age at the beginning of the research, with a similar weight and health condition as described [30]. A portable ultrasound machine (SSD-500; Aloka Co., Tokyo, Japan) was used together with a sector scanner transducer (7.5 MHz) and a needle guide. Briefly, after emptying the rectum and thoroughly cleaning the vulva and perineal area, the transducer was advanced to the external cervix. When the targeted follicles (≥2 mm in diameter) were stabilized on the puncture line, an 18-gauge needle was inserted in the guide, advanced through the vaginal wall and into the follicle antrum. Follicles (with follicular fluid), aspirated using continuous negative pressure (about 95 mmHg), were rinsed with D-PBS (Dulbecco's Phosphate-Buffered Saline, 14287072, Gibco, Grand Island, NY; pH 7.4) medium containing 3% BSA (Bovine Serum Albumin, A8806, Gibco) and 2 IU/ml heparin (000152-1, Solarbio, Shanghai). After recovery, the contents and flushing medium were filtered through an embryo filter (Fujihiraindustry Co., Tokyo, Japan) and COCs were recovered with the aid of a stereomicroscope.

### *In Vitro *Maturation

Oocytes were matured *in vitro *following the procedure described previously [32]. Briefly, COCs were cultured for 20 hr in TCM-199 (Gibco), which consisted of 10% fetal calf serum (FCS), 10 mg/ml LH, 1 mg/ml E2, and 1 mg/ml FSH, under a humidified atmosphere of 5% CO_2 _in air at 38.5°C.

### Preparation of Bovine Adult Fibroblasts (BAF) as Nuclear Donors

The BAF were isolated from the heifers' ear skin (heifers are identified as haplotype A and haplotype B by the mtDNA haplotype analysis) and washed several times in PBS containing 5% ABAM. BAF were cultured in 5 ml DMEM/F12 supplemented with 10% FBS in 100 mm^2 ^culture plate (Corning VWR) using tissue culture methods. The fresh bovine adult fibroblast cells were cultured for 3-6 passages and cryopreserved for future use. When desired, two- to five-passage bovine adult fibroblast cells were thawed and cultured for 3-5 days in DMEM/F12 (Gibco) containing 10% (v/v) FCS under a humidified atmosphere of 5% CO_2 _in air at 38.5°C. On the day of SCNT, immediately before donor cells were transferred into enucleated oocytes, the cells were dissociated by 0.25% trypsin-EDTA solution (Gibco Laboratories, Grand Island, NY). The cells were then washed by DMEM/F12 and treated by pronase 3 mg/ml (Calbiochem, Germany) for 50 sec, and washed 3 times and re-suspended with DMEM/F12.

### Nuclear Transfer, Activation, and Embryo Culture

After 20 hr maturation, only the metaphase II (MII) oocytes with first polar body, intact shape and uniform cytoplasm were used for enucleation. The glass pipettes used for oocyte holding, enucleating and injecting were prepared as described by Zhou [33]. Those oocytes to be used for nuclear transfer were stripped of cumulus cells by vortexing in Tyrode Lactate (TL-Hepes) +1 mg/ml of hyaluronidase for 5 min: 100 mM NaCl,3 mM KCl,0.27 nM L-glutamine, 6 mg/ml BSA (fatty acid free),1% basal minimum essential (BME) amino acid, and 1% minimal essential medium (MEM) nonessential amino acids [34]. Enucleation of MII oocytes was performed in 0.5 ml TL-Hepes in a chamber containing 1 ml of the medium, covered by mineral oil [34]. The chromosome removal was confirmed by Hoechst 33342 (10 μg/ml) staining under ultraviolet light. Enucleated oocytes were reconstructed with BAFs. Following reconstruction, the karyoplast-cytoplast complexes (KCCs) were placed in the 38.5°C preheated fusion medium (0.3 mol/L mannitol, 0.15 mmol/L Ca^2+^, 0.15 mmol/L Mg^2+^) for 2-3 minutes and then exposed to a double electric pulse of 2.5 kv/cm for 10 μs using the BTX Electro Cell Manipulator 2001 (BTX, San Diego) to initiate KCCs' fusion [33]. The contact surface between the cytoplast and the donor cell was parallel to the electrodes. The complexes were cultured in TL-Hepes, at 38.5°C for 30 to 60 minutes after the fusion pulse.(The complexes were cultured in ACM culture medium [100 mM NaCl, 3 mM KCl, 0.27 mM CaCl_2_, 25 mM NaHCO_3_, 1% BME amino acid and 1% MEM nonessential amino acids] [34], at 38.5°C for 2 hours after the fusion pulse.

About 2 hr after fusion, fusion rates were determined by visualisation. Then, the reconstructed embryos (fused KCCs) were replaced into 5 μM ionomycin for 4 min and then into in-house prepared additional culture of ACM culture medium with 10 μg/ml cycloheximide and 5 μg/ml cytochalasin B in four-well tissue culture plates (Nunclon, VMR) for 5 hr.

The reconstructed embryos were transferred into ACM culture medium supplemented with 1% FBS on mouse embryonic fibroblast (MEF) monolayer under humidified atmosphere of 5% CO_2 _in air at 38.5°C for 7 days. The embryos were changed to a new MEF plate with ACM supplemented with 10% FBS on the third day after the day of nuclear transfer which was set as day 0. The cleavage rates, 8-cell rates and blastocyst rates were determined at 48 hr, 72 hr and 7 days after activation, respectively.

### IVF and IVC Embryos

As a control, 20 hr after *in vitro *maturation, MII oocytes were fertilized with thawed bull semen from a single ejaculate [35]. The embryos were then cultured as described previously, and were collected at day 7. IVC embryos were obtained by perfusion and flush using physiological saline from bovine uterus at day 6.5 after fertilization, since *in vivo *embryos develop a little faster than *in vitro *embryos.

### Embryo Transfer

On the seventh day after observed estrus, the homotype and heterotype SCNT embryos were transferred nonsurgically into the uterine lumen ipsilateral to the corpus luteum of each heifer (Chinese yellow cattle). Pregnancies were confirmed on day 60 by ultrasonography and thereafter on day 90 by trans-rectal palpation. These cattle were observed periodically until the cloned calves were born.

### Indirect Immunoflurorescence of Histone H3K9

Procedures using an antibody to identify H3K9 dimethylation specifically in the context of constitutive heterochromatin in the embryo were performed as described by Santos et al. [6] with minor modifications. Briefly, blastocysts were washed in PBS, fixed for 15 min in PBS with 4% paraformaldehyde, and permeabilized with 0.5% Triton X-100 in PBS for 30 min at room temperature (RT). After washing with 0.05% Tween-20 in PBS, samples were blocked overnight at 4°C in blocking solution (1% BSA and 0.05% Tween-20 in PBS). The preimplantation embryos were stained overnight at 4°C with anti-histone3-dimethK9 antibody (Abcam, UK) diluted 1:250, followed by goat anti-rabbit-FITB-conjugated secondary antibody detection. Observations were performed with a Nikon TE2000 epifluorescence microscope. Embryos that could establish H3K9 asymmetry, with a hypomethylated trophectoderm and a hypermethylated ICM, were considered normal, whereas those embryos with a homogeneous staining pattern were classified as having aberrant H3K9 staining pattern [6,25].

### Preparation of Genomic DNA and Bisulfite Modification

DNA was isolated from three replicates of each of the sample types (*in vivo *embryo, IVF embryo, embryos derived from SCNT including homotype and heterotype haplotypes) as described previously [36]. Briefly, about 9-12 embryos for each group were suspended in lysis buffer (10 mM Tris-HCl, PH 8.0, 150 mM EDTA, 1% SDS and 100 ng/ml proteinase K). The mixture was incubated at 55°C for 20 min. Following two phenol/chloroform/isoamyl alcohol (50:49:1) extractions, the genomic DNA was precipitated in ethanol, pelleted, and redissolved in TE buffer (10 mM Tris-HCl, PH 7.6, 1 mM EDTA).

To analyze the methylation status of the *Oct4, Nanog *and *Sox2 *promoters, genomic DNA from the samples above were modified using methods described previously [37]. In brief, 1.5 μg of DNA was denatured in 50 μl of 0.2 M NaOH for 10 min at 37°C. Then, 30 μl of freshly prepared 10 mM hydroquinone (Sigma) and 520 μl of 3 M sodium bisulfite (Sigma) at pH 5.0 were added and mixed. The samples were overlaid with mineral oil to prevent evaporation and incubated at 50°C for 16 h. The bisulfite-treated DNA was isolated using Wizard DNA Clean-Up System (Promega). The DNA was eluted by 50 μl of warm water and 5.5 μl of 3 M NaOH were added for 5 min. The DNA was ethanol precipitated with glycogen as a carrier and resuspended in 20 μl of water. Bisulfite-treated DNA was stored at -80°C until ready for use.

Converted DNA was amplified using specific primers designed with MethPrimer software http://www.urogene.org/methprimer/index1.html. The primers used for PCR amplification are presented in Table 5.

**Table 5:** Primers for pyrosequencing analysis.

Genes	PCR primers(5'-3')	Length (bp)	CpG site
*Oct-4*	ATTTGGATGAGTTTTTAAGGGTTTT	292	23
	ACTCCAACTTCTCCTTATCCAACTT		

*Nanog*	TTTTTTAATTATAATTTGATGGGGT	288	13
	CTAACACACCTTAAATAAACAAACC		

*Sox2*	GGGATATGATTAGTATGTATTTTTT	230	15
	TTCTCCATACTATTTCTTACTATCCTCC		

The amplicons of development-related genes promoter region derived from each sample were cloned into pMD-18T vector (TaKaRa, China). Individual clones were purified and then sequenced.

### Statistical Analysis

All data were obtained from at least four replicates. Continuous data were analyzed with Student's *t *test (P < 0.05) and proportional data such as potential differences for embryo development or promoter DNA methylation profiles among different groups were analysed with Chi-square tests (SAS, Version 6.12; SAS Institute, Cary, NC). Differences with a P-value < 0.05 were considered significant.

## Authors' contributions

FYZ, YTZ and SZH designed the study, while ZHY, YYZ, FY, JF, LWZ and PFG carried out the experiments (ZHY performed the DNA methylation. YYZ, JF and FJ carried cell culture and H3K9 methylation, LWZ, PFG carried out SCNT experiments) ZHY, YYZ, JF, YTZ and FYZ prepared the manuscript. All authors read and agreed with the final draft.
